# Which tibial implantation site for the deep medial collateral ligament should be chosen to control anteromedial rotatory instability of the knee?

**DOI:** 10.1002/jeo2.70585

**Published:** 2025-12-07

**Authors:** Antoine Hamon, Harold Common, Théo Cojean, Henri Robert

**Affiliations:** ^1^ Orthopaedic Department University Hospital Rennes France; ^2^ University Claude Bernard Lyon 1 France; ^3^ Orthopaedic Department Hospital Haut Anjou Château‐Gontier‐sur‐Mayenne France

**Keywords:** anteromedial bundle, biomechanic, Dyneelax® laximeter, medial side injuries, medial side plasty

## Abstract

**Purpose:**

Conventional techniques (medial collateral ligament + posterior oblique ligament reconstructions), such as those by Lind and LaPrade, do not fully restore native knee stability in severe medial injuries. This study aimed to determine the optimal tibial insertion site for an anteromedial (AM) reconstruction strand mimicking the deep medial collateral ligament (dMCL), to better control anteromedial rotatory instability (AMRI) in knees with complete medial injury (sMCL + dMCL + POL).

**Methods:**

Twenty fresh‐frozen cadaveric knees (13 female, 7 male donors; mean age: 80 years) were tested using Dyneelax® static laximeter at 30° of flexion. After standardised transections of the superficial MCL (sMCL), deep MCL (dMCL), and posterior oblique ligament (POL), each knee was reconstructed using a trifid flat graft including an AM strand. Knees were assigned to two groups based on the tibial insertion angle (α) between the AM strand and the sMCL: group *α* ≤ 20° (anatomical reconstruction) and group *α* > 20° (oblique isometric reconstruction). Anterior tibial translation (ATT), internal rotation (IR), and external rotation (ER) were measured under 200 N of force and 5 N‐m torques. Residual laxities were calculated in both absolute and relative terms, compared to the intact state, and analysed using ANOVA and Student's t‐tests.

**Results:**

Both reconstruction techniques significantly reduced laxity compared to the transected state. However, group *α* > 20° showed significantly lower residual laxity for ATT (0.74 ± 0.58 mm vs. 1.30 ± 0.56 mm, *p* = 0.04) and for ER (0.35 ± 0.39° vs. 1.05 ± 0.89°, *p* = 0.04), with no significant difference for IR (*p* = 0.24).

**Conclusion:**

There is a trend toward better stability with a more oblique AM strand, which must be confirmed by further biomechanical studies. This trifid flat graft approach more accurately replicates the biomechanical function of the dMCL and anteromedial capsule, and could provide a refined strategy for reconstructing severe medial knee instability

**Level of Evidence:**

Level V, experimental cadaveric study.

**Abbreviations**
ACL: anterior cruciate ligament
AM: anteromedial bundle
AMC: anteromedial capsule
AMRI: anteromedial rotatory instability
dMCL: deep medial collateral ligament
MCL: medial collateral ligament
PMC: posteromedial capsule
POL: posterior oblique ligament
sMCL: superficial collateral ligament

## INTRODUCTION

Medial side knee injuries are frequently under‐estimated when associated with anterior cruciate ligament (ACL) ruptures. Willinger et al. reported that 67% of presumed ‘isolated ACL injuries' had a lesion to the medial side (medial collateral ligament, posteromedial complex and medial meniscus) of the knee [37]. The lack of recognition of these injuries termed ‘the hidden enemy' by Malinowski et al. places an additional strain on the ACL reconstruction and increase the rate of ACL graft failure [26, 27, 33]. These injuries occur during valgus and rotatory movement of the tibia in sports such as soccer, skiing, and martial arts among others and in high‐velocity injuries [35]. According to the Hughston classification, later revised by Fetto and Marshall, the Grade I and II injuries usually respond well to various nonoperative management [18, 19, 34]. Grade III lesions (MCL + POL injuries) characterised by valgus laxity at both 0° and 30° of knee flexion and excessive internal and external rotation, may require surgical procedures in cases of multiligament injuries [19].

The anatomy and functions of each of these structures have been described extensively over time and confirmed recently [2, 3, 18, 19]. The superficial medial collateral ligament (sMCL) is a flat structure with fan‐like fibres than span a wide femoral origin and tibial insertion. The sMCL is the primary passive restraint to valgus stress and an important restraint to tibial external rotation. A previous study highlighted that different zones of the broad native MCL (anterior, intermediate, and posterior third) perform different functions [3]. The deep medial collateral ligament (dMCL) is a major restraint to external tibial rotation (ER) near knee extension [3]. The posterior oblique ligament (POL) is a primary restraint to valgus and internal tibial rotation (IR) in extension but does not play a significant role in anteromedial rotatory instability (AMRI) [19, 24, 32]. The antero‐medial capsule and the sMCL are involved in rotational control from 30° of flexion onward [3, 18, 21].

Two techniques, often seen as the ‘gold standard’, for addressing medial side rotatory and frontal laxity, were proposed by Lind et al. in 2009 and LaPrade et al. in 2012, both of which involve reconstruction of the sMCL and the POL [23, 24]. However, laboratory testing has indicated that these techniques do not always restore native knee stability because they neglected the importance of the dMCL as a primary restraint to external tibial rotation and of the anteromedial capsule (AMC) [6, 18, 27].

New MCL reconstruction techniques are being proposed to better restore the dMCL in addition to the sMCL reconstruction. Some surgeons have proposed reinforced reconstructions of the sMCL and dMCL in the form of a ‘short isometric construct’ in AMRI [7, 8, 29]. A reconstruction of an oblique and isometric anteromedial strand (AM) to reproduce the function of the dMCL and the anteromedial capsule, associated with a reconstruction of the sMCL and the POL have already been proposed but not biomechanically evaluated [29]. Moreover, because the native MCL is a broad and flat structure, its contributions to ER, IR and valgus control are not fully reproduced by round tubular grafts alone [6]. Biomechanical studies have demonstrated the advantages of flat reconstruction techniques to improve restoration of the native knee kinematics functions [13, 29].

The objective of the present work was to search for the ideal tibial site for distal tibial fixation of the AM strand, which to our knowledge, has not yet been determined. Two tibial positions of the AM reconstruction according to the angulation α between the AM simulating the dMCL and the sMCL were evaluated. The first AM group with a slight forward oblique inclination, less than 20°, according to anatomic works (Group *α* ≤ 20°) and the second AM group with inclinaison more than 20° (Group *α* > 20°), realising an isometric and non‐anatomic reconstruction to achieve better control of ATT and ER [2, 9, 30, 36].

It was hypothesised that increasing the divergence between the anterior strand and the sMCL may improve the control of ATT, IR and ER induced by transections of the medial plane resulting in a grade III medial injury.

## MATERIALS AND METHODS

### Specimen preparation

Twenty non‐paired, fresh‐frozen cadaver lower limbs from 13 female and 7 male donors (mean age: 80 years; range, 71–91 years) were tested in the Anatomy Laboratory of the Medical School of Rennes, France. Written consent (donation to university anatomy programme) from the donor for their use for educational and research programmes was obtained for each specimen. The lower limbs were collected by disarticulation at the hip and kept frozen at −20°C before the tests. All specimens were thawed at room temperature (20°C) for one day before use. The knees were mobilised 10 times in flexion, extension and rotations to ensure that they were flexible and able to flex to at least 130°. The knees were stable for clinical testing in frontal and sagittal planes. Inclusion criteria were stable and mobile knees, and exclusion criteria were signs of ligamentous injuries, bone anomalies, osteoarthritis, or scars indicating previous surgery. All the knees had an X‐ray (coronal and sagittal planes) before dissection and were classified according to Ahlbäck classifications (Grades I to IV). One knee initially selected was rejected (Ahlbäck grade > I) by the surgeon who performed the dissections.

### Dissection technique

Two orthopaedic knee surgeons (HR and HC) performed all dissections and reconstructions in the same standardised way to ensure reproducibility. A medial arc‐shaped incision was made from the medial epicondyle to the pes anserinus to expose the medial side. The skin and subcutaneous tissue were removed, leaving the ligaments, joint capsule and musculature intact. The medial aspect of the knee was divided into three parts. The identification starts in the middle third as it represents the MCL divided into superficial (sMCL) and deep layers (dMCL). The anterior third, named anteromedial retinaculum, was left intact. In the posterior third, the localised thicker band in the posteromedial capsule (PMC) is the POL. No. 2 braided wires sutures were tied around both tested structures: sMCL + dMCL and PMC, to facilitate their secondary transection.

**Figure 1:**
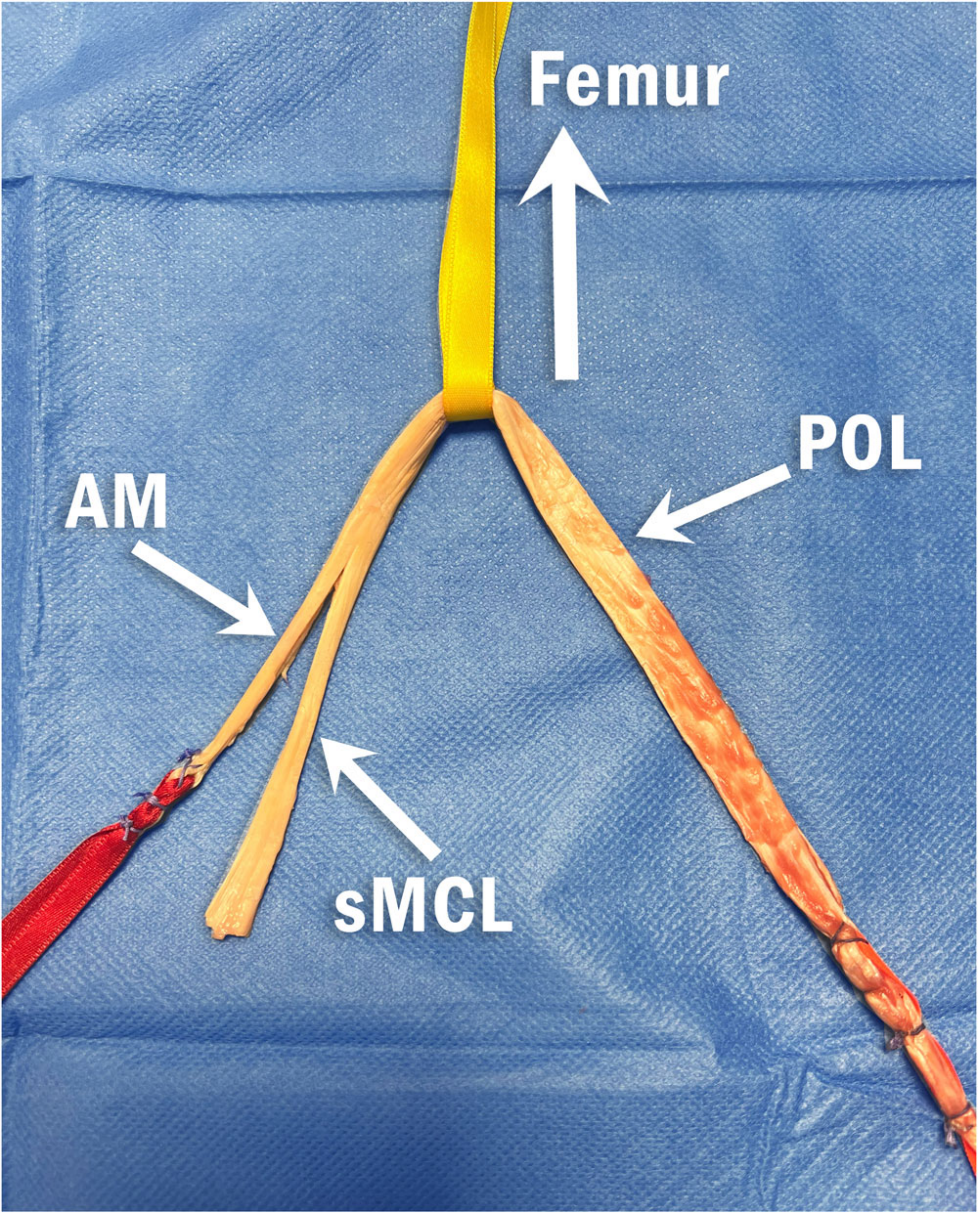
The allograft tendon was divided into two bundles: approximately two‐thirds of the width for the sMCL and one‐third for the AM in the anterior bundle and the posterior bundle. The graft has a “Trifid flat configuration” with an anterior bundle for the AM bundle, an intermediate bundle for the sMCL and a posterior bundle for the POL. AM, anteromedial bundle; dMCL deep superficial medial collateral; MCL, medial collateral ligament; POL, posterior oblique ligament; sMCL, superficial medial collateral ligament.

**Figure 2:**
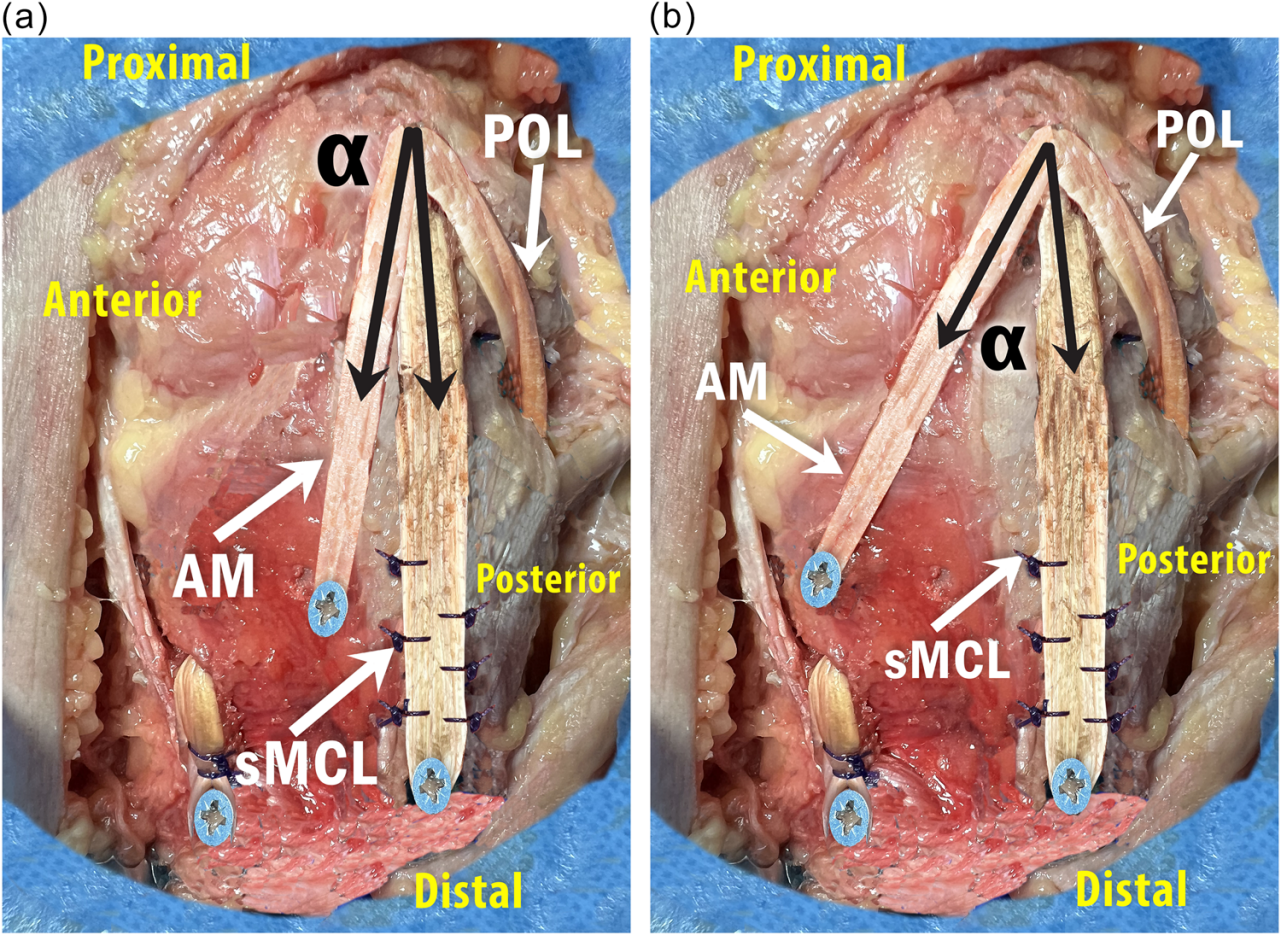
(a) Group *α* ≤ 20°. Medial view of a right knee. The sutured loop of the allograft was inserted into the femoral tunnel and fixed after transection with a metallic bicortical screw at the lateral femoral cortex (*Epicondyle). The tibial sMCL tunnel was 60 mm below the joint line at the centre of the anteroposterior width of the native sMCL The AM tibial insertion is located 20 mm below the joint line and 10 mm in front of the anterior edge of the sMCL. Isometry of the AM is sought with a double point compass between the epicondyle and the AM insertion site, while the knee was taken through a full range of motion. The divergence angle α is measured between the dMCL and the sMCL and range from 10° to 20°. The AM bundle was fixed with a bicortical tibial screw. The sMCL (sMCL, superficial medial collateral ligament; dMCL, deep medial collateral ligament; AM, anteromedial bundle). (b) Group *α* > 20°. Medial view of a right knee. The AM tibial insertion is located 20 mm below the joint line and 30 mm in front of the anterior edge of the sMCL. The divergence angle α is measured between the dMCL and the sMCL and range from 20° to 40°. AM, anteromedial bundle; dMCL, deep medial collateral ligament; sMCL, superficial medial collateral ligament.

The tibialis anterior tendon was harvested over a length of approximately 20 cm via an anterior approach to the foot using an open stripper. The tendon was divided into two bundles: approximately two‐thirds of the width for the sMCL and one‐third for the dMCL. The graft has a trifid configuration with an anterior bundle for the dMCL, an intermediate bundle for the sMCL and a posterior bundle for the POL (Figure 1). The tibial sMCL tunnel was 60 mm below the joint line at the centre of the anteroposterior width of the native sMCL. The sMCL was secured by a bicortical screw (diameter of 5 mm). A single femoral insertion for the MCL and the POL was identified proximal and posterior to the apex of medial epicondyle. The dMCL tibial insertion is located 20 mm below the joint line and 10 mm (Group 'anatomic' with an angle *α* ≤ 20°) or 30 mm (Group isometric with an angle *α* > 20°) in front of the sMCL (Figure 2a,b). Isometry of the dMCL construct in the Group *α* > 20° is critical and was assessed using a double point compass (FH Orthopedics, Heimsbrunn, France) to avoid the need of two K‐wires and a thread between the two wires [14, 29]. A 2.4 mm eyelet pin was drilled into the femoral pilot hole bicortically and oriented 40° anteriorly and proximally. A 4.5 mm cannulated drill is used to create a femoral bicortical tunnel, and then a 30‐mm deep femoral socket is drilled at a diameter matching the graft loop. The graft loop is pulled into the femoral socket and fixed at the tunnel exit with a screw. During testing, specimens were kept moist with water.

### The Dyneelax® laximeter

**Figure 3:**
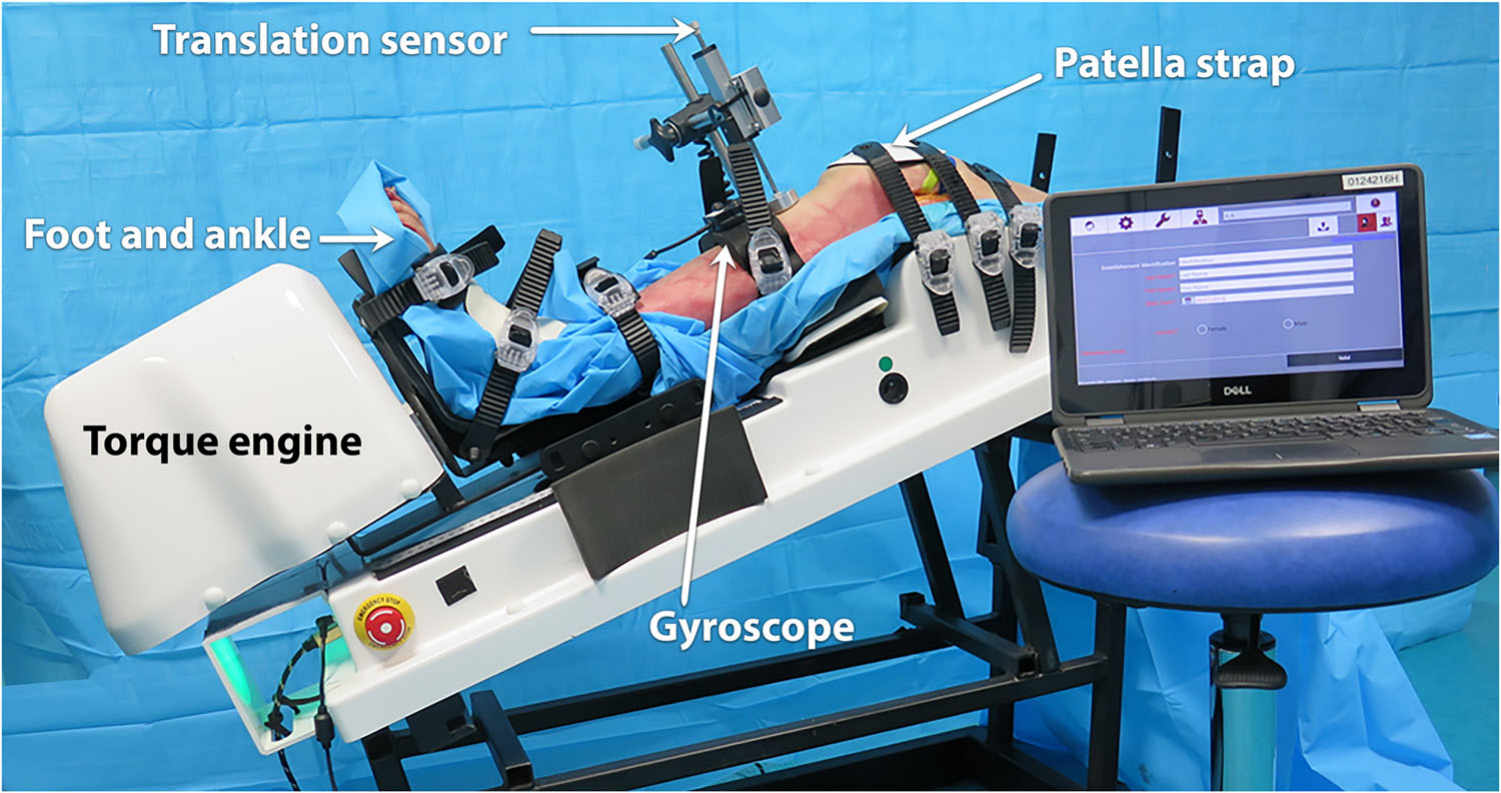
The Dyneelax® allows ATT under loads up to 250 N and tibial rotation (IR and ER) up to 8 N‐m separately. The lower limb was placed on a thermoformed support at 30° of flexion. The femoral head was secured horizontally by a transverse rod. The foot and ankle were attached to a dual bootstrap, providing a stationary block under the tibia. ATT is produced by a linear jack and axial torque ([IR and ER]) by a rotation engine. ATT was registered by a translation sensor and rotations (ER and IR) by a gyroscope. ATT, anterior tibial translation; ER, external rotation; IR, internal rotation.

A non‐invasive and static translational and rotational knee laximeter, the Dyneelax® was used (Genourob Company, Laval, France) (Figure 3). The system allows for repeatability of motion within ± 0.1 mm in translation and ± 0.1° in rotation [11]. Dyneelax® allows anterior tibial translation (ATT) under forces up to 200 N and rotation torque up to 5 N‐m independently from the translation, but no valgus stress. The lower limb was placed on a thermoformed support at 30° of flexion (only angle permitted by the laximeter). The femoral head was secured horizontally by a transverse rod (diameter 8 mm) to prevent any rotation of the femur. The foot and ankle were attached to a dual bootstrap, providing a stationary block under the tibia. The initial knee position was defined by ‘the patella at the zenith’ and the foot‐ankle block was in a natural resting position of the leg controlled by the absence of constraint on the boot sensors. The initial position of the lower limb was not further modified during all the testing conditions. Therefore, any change in translation or rotations was due to the surgical alteration of the joint. The induced ATT and rotations were measured from this neutral position and were recorded. All specimens were submitted to the same forces (anterior drawer force of 200 N) and secondarily to the torques (5 N‐m of IR and ER).

### Testing protocol

Knees were randomly allocated to the *α* ≤ 20° or *α* > 20° reconstruction technique (10 knees in each group). Knee laxities were registered in the three following states: intact knee, sMCL + dMCL + PMC transection, and finally, reconstruction with the AM strand using either the *α* ≤ 20° or *α* > 20° configuration. The ligament sections above the medial meniscus (to create the completely MCL + POL deficient state) were performed with the help of size two‐braided wire, doubled up and used in the same manner as a Gigli saw. To account for any viscoelastic effects of the tissues, all measurements were recorded five times, and the mean data were taken as the result in each test. The results were registered as translation curves (in millimetres) and rotation curves (IR and ER laxity) in degrees (absolute values) of the tibia relative to the femur after the transection of the ligaments, and thereafter the reconstructions with one randomly chosen technique (Group *α* ≤ 20° and Group *α* > 20°). Residual laxities calculations were based on two formulas:

* Residual laxity (mm) = Laxity Reconstructed – Laxity Intact
* Residual laxity (%) = Laxity Reconstructed – Laxity Intact/Laxity Transected – Laxity Intact × 100.

### Statistical analysis

Statistical analyses were performed with JASP statistical software (version 0.19.3; University of Amsterdam). The sample size was calculated post‐hoc by power analysis with G*Power Version 3.1.9.7 (Heinrich Heine University). Laxity measurements at 30° of knee flexion were used, as reported in two previous laboratory and controlled studies on medial‐side reconstruction [18, 30]. A sample size of eight specimens per group was determined to be sufficient to detect a statistically significant difference of 2° in internal and external rotation, and 2 mm in translation, with a statistical power of 80% and a confidence level of 95%. Based on our experience, a small number of cadaveric specimens can be excluded due to unanticipated degenerative changes or bone fragility (age of specimens). For this reason, we intentionally designed the sample to reach 20 knees.

To exclude baseline bias, the laxity induced by sectioning itself between groups was compared.

For each knee, Δ Transection = Laxity Transected – Laxity Intact, was computed for ATT, IR and ER, and then compared between the *α* ≤ 20° and *α* > 20° groups using two‐sample T‐tests.

The laxity data were tested for normal distribution with the Shapiro–Wilk test, and the homogeneity of variances was evaluated with the Brown–Forsythe test, given the small sample size of each group. First, results were compared between each state of transection and reconstruction for both group ≤ 20° and group > 20° for AM bundle reconstruction using repeated measures one‐way analysis of variance (ANOVA) with a post hoc Bonferroni correction for multiple t tests comparisons.

Residual laxities were then established after the reconstruction compared to the intact state of the knee according to the formula described above. They were expressed first in millimetres for translation and degrees for rotations. Student's T‐tests were used to compare group ≤ 20° and group > 20° to assess which angulation α is better for AM bundle reconstruction to restore greater anterior translation, internal, and external rotational stability. A *p*‐value < 0.05 was considered statistically significant.

## RESULTS

**Figure 4:**
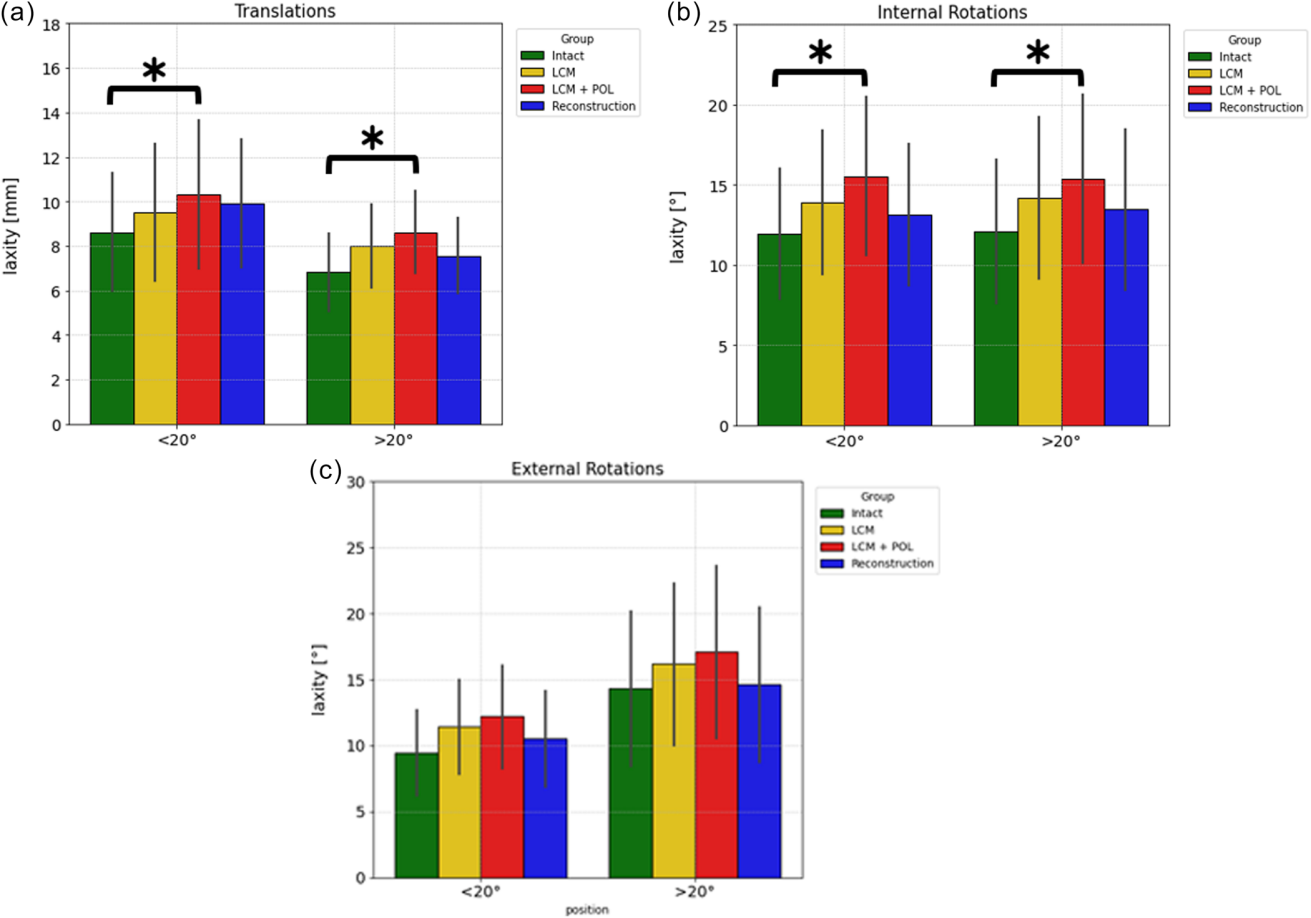
(a) Data presenting the laxities, expressed as mean ± SD, measured at 200 N for ATT tests. Results are presented after each transection and after the reconstruction for both groups: AM strand at *α* ≤ 20° and at *α* > 20°. Statistically significant differences compared with the previous states are indicated by an asterisk * (*p* < 0.05). (b) Data presenting the laxities, expressed as mean ± SD, measured at 5 N‐m for IR. tests. Results are presented after each transection and after the reconstruction for both groups: AM stand at *α* ≤ 20° and at *α* > 20°. Statistically significant differences compared with the previous states are indicated by an asterisk * (*p* < 0.05). (c) Data presenting the laxities, expressed as mean ± SD, measured at 5 N‐m for ER. tests. Results are presented after each cutting state and after the reconstruction for both groups: AM stand *α* ≤ 20° and *α* > 20°. Statistically significant differences compared with the previous states are indicated by an asterisk * (*p* < 0.05). AM, anteromedial; ATT, anterior tibial translation; ER, external rotation; IR, internal rotation; SD, standard deviation.

Laxities at 200 N for ATT and at 5 N‐m for IR/ER in the intact state, post‐transection states (MCL then MCL + POL), and reconstruction states for both groups are summarised in Table 1 and Figure 4a–c.

**Table 1:** Laxities at 200 N for anterior translations and at 5 N‐m for internal/external rotations, for intact state (intact), transected states (MCL and MCL + POL) and reconstruction state between Group *α* ≤ 20° and Group *α* > 20°.

		Gr *α* ≤ 20°	Gr *α* > 20°	*p*‐Values
Anterior translation (mm)	Intact	8.62 ± 2.65	6.82 ± 1.72	0.007 *
	MCL sec.	9.52 ± 3.07	8 ± 1.82	0.044*
	MCL + POL sec.	10.31 ± 3.31	8.62 ± 1.83	0.015*
	Reconstruction	9.92 ± 2.87	7.56 ± 1.68	<0.001*
Internal rotation (°)	Intact	11.92 ± 4.05	12.08 ± 4.48	ns
	MCL sec.	13.91 ± 4.48	14.2 ± 5.03	ns
	MCL + POL sec.	15.53 ± 4.92	15.36 ± 5.25	ns
	Reconstruction	13.15 ± 4.39	13.48 ± 4.99	ns
External rotation (°)	Intact	9.47 ± 3.22	14.32 ± 5.84	<0.001*
	MCL sec.	11.45 ± 3.54	16.19 ± 6.11	<0.001*
	MCL + POL sec.	12.21 ± 3.89	17.09 ± 6.5	<0.001*
	Reconstruction	10.54 ± 3.6	14.64 ± 5.85	0.001*

Abbreviations: MCL, medial collateral ligament; POL, posterior oblique ligament.

Mean Δ Transections (Laxity Transected – Laxity Intact), which represent instability induced by MCL + POL transection, are presented in Table 2. Sectioning induced similar instability across groups (Δ Transection), all non‐significant. Table 3 shows that reconstruction yielded superior control of ATT and ER with *α* > 20° with significant differences: there is a Δ of 0.564 mm for ATT, −0.5° for IR and 0.7° for ER. Table 3 more clearly highlights the difference between groups. This normalises the reconstruction effect to the lesion‐induced laxity of each specimen, making proportional effects comparable across heterogeneous knees. There is a Δ of 32.7% for ATT (*p* = 0.01), −15.6% for IR (*p* = 0.09) and 20.9% (*p* = 0.02) for ER between groups ≤ 20° and > 20° (Table 3).

**Table 2:** Mean Δ transection (Laxity Transected – Laxity Intact), which represents laxity induced by MCL + POL transection, were compared using Student T‐tests.

	Gr *α* ≤ 20°	Gr *α* > 20°	*p*‐Values
Δ transection anterior translation (mm)	1.68 ± 0.87	1.8 ± 0.82	0.5
Δ transection internal rotation (°)	3.6 ± 142	3.27 ± 1.12	ns
Δ transection external rotation (°)	2.74 ± 1.18	2.77 ± 1.61	ns

*Note*: Sections induced similar laxities across both groups (Group *α* ≤ 20° and Group *α* > 20°), all were non‐significant.

Abbreviations: MCL, medial collateral ligament; POL, posterior oblique ligament.

**Table 3:** Residual laxities in mm, in degrees and in % for anteromedial (AM) stand reconstruction in Group *α* ≤ 20° and Group *α* > 20° compared to the intact states at 200 N for anterior translations (mm) and at 5 N‐m for internal/external rotations (°).

	Gr *α* ≤ 20°	Gr *α* > 20°	*p*‐Values
Anterior translation	1.30 ± 0.56 mm	0.74 ± 0.58 mm	0.04*
	73.45 ± 27.82%	40.68 ± 24.53%	0.01*
Internal rotation	1.19 ± 0.81 mm	1.70 ± 1.04 mm	0.04*
	33.16 ± 18.74%	48.82 ± 20.77%	0.09
External rotation	1.05 ± 0.89 mm	0.35 ± 0.39 mm	0.04*
	36.34 ± 22.07%	15.43 ± 14.47%	0.02*

*Note*: Both reconstructions were compared using a Student T‐test. Statistical significance was set at *p* < 0.05.

## DISCUSSION

The main finding of this cadaveric biomechanical study on trifid reconstruction was that residual laxity in absolute values was significantly lower in ATT and ER in the Group *α* > 20° than in the Group *α* ≤ 20°. Both techniques with an AM bundle can reduce most of the laxity induced by a Grade III injury on the medial side (sMCL + dMCL+ POL) in IR and ER, but to a lesser extent in ATT. The ACL reconstruction, possibly in conjunction with other procedures, will primarily take control of the anterior translation. In relative value, residual laxity was always lower in translation and rotation in the Group *α* > 20° technique compared to the Group *α* ≤ 20° technique, with statistically significant difference (Table 2). In both techniques, there were some residual laxities in ATT, ER and IR, but significantly less in the Group *α* > 20°, thus no reconstruction technique completely restores the knee's native stability. These results could confirm the study's hypothesis. In a previous laboratory study, Guegan et al. showed that the transections of the MCL and POL significantly increased ATT and rotations [18]. Because Δ Transection (Laxity Transected – Laxity Intact) did not differ between groups for ATT, IR or ER, the sectioning procedure itself did not induce greater baseline laxity in one group. Therefore, the significant post‐reconstruction differences observed for ATT and ER reflect the effect of the tibial insertion technique (*α* > 20°) rather than pre‐existing laxity group differences.

Isolated anatomical sMCL reconstruction or sMCL plus POL reconstruction are unable to restore ATT, valgus rotation, and ER at 30°, 60°, and 90° of flexion to the intact state, due to the complex functionality of the broad and flat sMCL [4, 12, 19, 26]. The two most widely used medial reconstruction methods are the Lind technique and the LaPrade technique, both of which reconstruct the sMCL and the POL, neglecting the dMCL [23, 24]. These techniques have primarily been evaluated postoperatively for controlling valgus, but less so for restoring rotation stability. Therefore, the POL reconstruction may be beneficial for patients needing IR restrain in full extension (posterior cruciate ligament + POL injuries in posteromedial rotatory instability) but may not be useful for patients with only AMRI. In the anatomic LaPrade technique we need two double‐strand grafts to reconstruct the sMCL and POL with four tunnels and four fixation implants (two in the femur and two in the tibia) [23]. In the Lind technique, the tibial attachment of the semitendinosus is preserved, which results in a non‐anatomic and anisometric reconstruction of the MCL, which has been reported to have poorer outcome even in valgus stress [15]. Recently a new anteromedial reconstruction technique has been developed by Lind et al. [25]. An AM reconstruction is added to provide a brake against the AMRI. The semi tendinosus is left attached to the tibia and fixed proximally and posteriorly to the femoral epicondyle with an interference screw. The free end of the ST is then directed distally and fixed in the centre of the tibial sMCL with an interference screw. To reconstruct the dMCL tibial insertion, the anterior strand is fixed to the tibial below the joint line with a suture anchor. The addition of an AM graft to the sMCL reconstruction improved control of ATT, ER and valgus relative to the intact state at all flexion angles [4, 16]. A triple strand technique that can reproduce the function of the dMCL, sMCL, and POL better restore the frontal and rotational stability than a double strand [27]. In clinical practice, autogenous hamstring tendon grafts, are frequently harvested but they are active stabilisers against valgus and ER, thus their sacrifice may be questioned for MCL reconstruction [22]. Borque et al. and Shatrov et al. suggested a short oblique bundle and the use of synthetic tapes for the dMCL reconstruction as support to protect the soft tissue, while the MCL heals [9, 31]. The use of synthetic ligament (polyester tape) can be of concern given the high stiffness of the synthetic material in case of poor isometry and the short‐term results available [7, 9].

**Figure 5:**
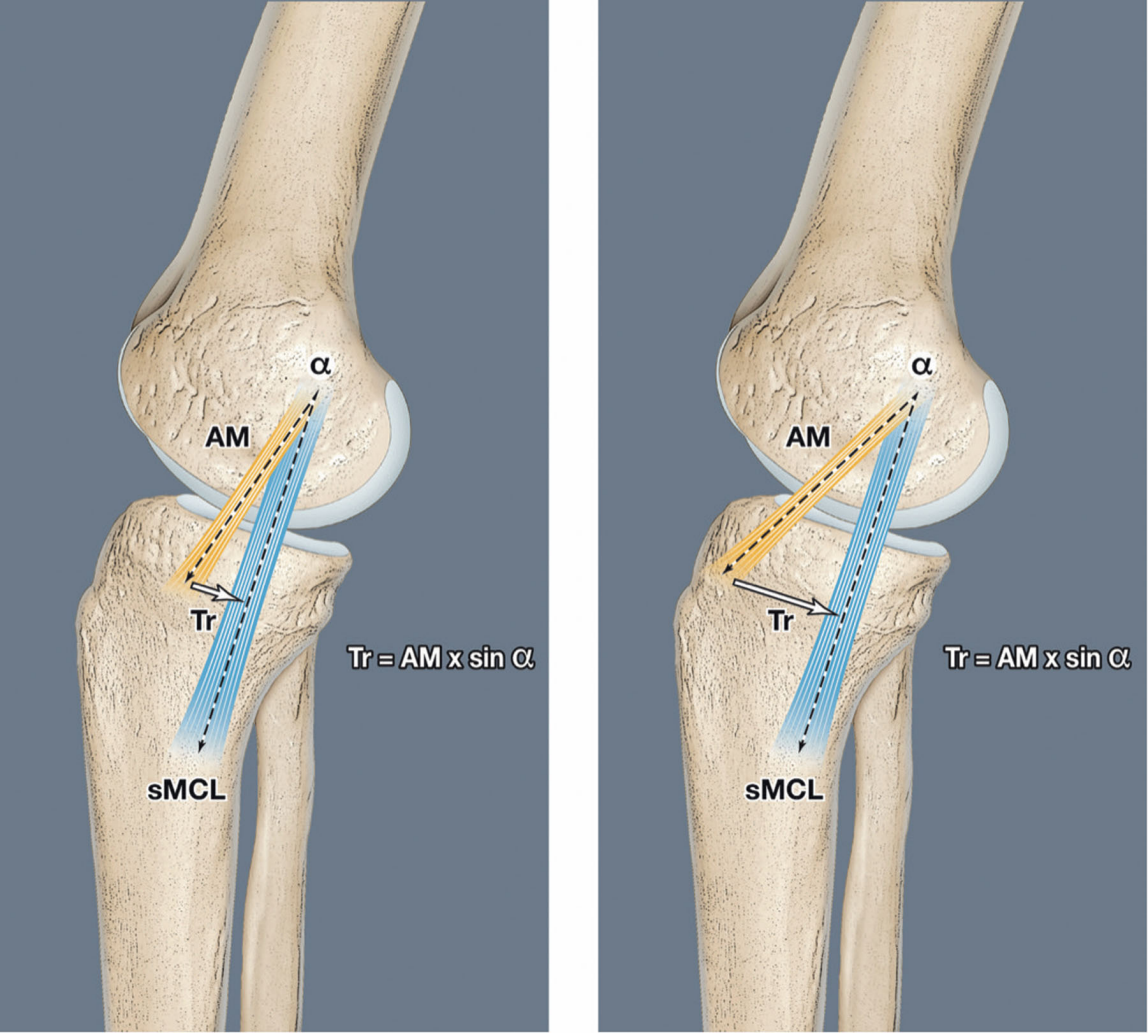
(a, b) Diagrams a and b show the changes in the length of the vector Tr as a function of the angle α according to the formula: Tr = dMCL × sinus *α*. If the angle increases from 15° to 30°, the vector Tr doubles in length, which means that the brake on the ATT and the ER significantly increases. AM, anteromedial; ATT, Anterior tibial translation; dMCL, deep medial collateral ligament; ER, external rotation; sMCL, superficial medial collateral ligament.

A critical technical step for any MCL reconstruction is determining the correct femoral tunnel position to ensure graft isometry [5, 15]. A slight misplacement of the femoral site can significantly affect graft length behaviour (over tension or slackening of the graft). Placing the sMCL femoral attachment site in the middle or posterior part of the native sMCL femoral attachment site gives a more favourable isometry [4, 26]. The position of the tibial attachment more‐or‐less anterior to the sMCL is the key point described in the present work. The angle α between the sMCL and the AM should be > 20° providing an oblique graft orientation that better mimics the function of the dMCL. A previous biomechanic study suggested that this more oblique orientation of the AM reconstruction is advantageous in restraining ER [3]. Gellhaus et al. tested three different tibial attachment of the AM and did not find a better effect on efficacy with one specific site [16]. The present study specified the most effective isometric implantation site for the AM strand on the tibia epiphysis. A more horizontal orientation (*α* ≥ 20°) of the AM bundle provides a stronger horizontal vector (brake on tibial anterior shear force) than a more oblique implantation (*α* ≤ 20°). Figure 5a,b show the changes in the length of the vector Tr as a function of the angle α according to the formula: Tr = dMCL × sinus *α*. If the angle increases from 15° to 30°, the vector Tr doubles in length, which means that the brake on the ATT and the ER significantly increases. Griffith and al. suggested that surgeons should be cautious not to over tension the AM strand, as this might lead to some over constraint on the knee [17]. The technique using an oblique and isometric strand at ≥ 20° (average angulation of 33° in our series) helps to compensate for the rupture of the dMCL and the anteromedial capsule (AMC). The main function of the dMCL is to control the ER at 0°–30° of flexion. In the last few years, the description and the function of the AMC were reported in the control of anterior translation and rotations [20]. An anterior oblique ligament (AOL) was identified recently in the AMC, originating anterior and distal to the medial epicondyle and inserted at 1 cm below the joint line, anterior to the sMCL and extra capsular. The AOL is in tension during combined flexion and external rotation of the tibia. These two structures (dMCL + AOL) in the anterior one‐third of the medial side of the knee may be damaged in valgus‐external rotation of the knee [14]. The AM bundle of a trifid reconstruction could therefore also help restore the function of the AOL.

Biomechanically, flat grafts can better distribute load and accommodate length changes (isometry) across knee motion. A flat graft aims to replicate the native footprint and shape of the sMCL, whereas a conventional tubular graft is unable to distribute loads and accommodate length changes across knee motion. The single‐bundle round graft fail to fully restore valgus and rotational stability at mid‐range knee flexion angles, leaving residual laxity [4]. Petersen et al. and Aberman et al. described a flat graft technique transforming a hamstring tendon into a ‘ribbon‐like’ structure replicating the native MCL breadth to promote tendon‐to‐bone healing [1, 28]. Deichsel et al. confirmed, in a robotic study, that flat reconstruction of the sMCL and anteromedial structures restored native knee kinematics more effectively than round grafts [13]. In a flat tendon, tension can be redistributed across the graft at different flexion angle [1]. Briese et al. compared the biomechanical and anatomical properties of several flat grafts (semitendinosus, gracilis, quadriceps tendons and iliotibial band) prepared in single and double‐strand grafts. SemiT‐single‐strand and quadriceps appear to better mimic the biomechanical properties (stiffness) and cross‐sectional area (mm^2^) of the native sMCL [10]. These data suggest that using a flat graft could improve rotational stability and promote better biological integration through increased contact surface between the graft and the native ligament surface. This concept aligns with fundamental biomechanical principles stating that the graft's morphology should ideally replicate the shape and orientation of the injured ligament to effectively restore its function [10, 12].

For symptomatic chronic valgus instability with medial gapping and rotatory instability, surgical management is the method of choice and involves reconstruction of the median stabilisers: the dMCL, the sMCL and the POL. The extent of the reconstruction depends on the clinical examination in pre‐op (valgus in extension, anteromedial drawer test, dial test…), the analysis of the MRI, the laximeter testing and finally in the operating room under anaesthesia. In cases of extensive medial injury (sMCL + dMCL + POL), Robert et al. described a reconstruction technique using a trifid and flat graft, based on the conclusions of this study [29]. In case of valgus and AMRI, the POL reconstruction may not be required, and a sMCL + dMCL reconstruction will be sufficient in such a situation [27]. The ACL was left intact, so this work only studied the reconstruction of the MCL and POL, excluding variability among ACL reconstructions. Additional transection and reconstruction of the ACL might be closer to clinical situations, but the interpretations of the results may be more difficult. All measurements were performed objectively using the same Dyneelax® laximeter, without any mobilisation after its initial setup [10]. The position of the knee in flexion 30° was not changed after each stage (transection then reconstruction).

Our study has several limitations. Specimen age and reduced bone quality may have negatively affected the rigid fixations of the reconstructions. The average age of the donors was not very close to the age of the population targeted by the applications of this study. To decrease the risk of graft slippage, in porotic bones, the grafts were fixed with bicortical screws (diameter of 5 mm) on both the femur and the tibia. For each knee, only 20 repetitive tests at 30° of flexion were performed, without modification of the position, which avoids the potential for failure of the fixations, slippage or stretching of the grafts. Some authors reported the need for graft re‐tension before proceeding with the next flexion angle for testing [27]. We excluded re‐tension of the fixations due to the high stability of the bicortical screw fixations and the risk of overcorrection they might cause. The present study concerns only reconstruction of the passive medial ligamentous restraints, excluding the dynamic stability provided by the surrounding muscles (semimembranosus and hamstrings tendons). Sims et al. reported that the most common abnormality found during AMRI surgery was the rupture of the semimembranosus tendon attached to the posteromedial capsule [31]. The loads applied were not as high as those used during sports activities but were like those used during clinical examination. Higher loads have the potential to stretch the remaining intact structures leading to uncontrolled ligament and joint laxities. A tensioning device or other force gauge were not used to standardise the amount of initial tension (usually 60 N) on the grafts, and thus there may be bias introduced by different initial manual tensions. The graft tension on the femoral loop in line with the femoral tunnel was always performed at 30° of flexion. The knees were tested only at 30° of flexion for the total arc of flexion (0°–90°) was impossible with the Dyneelax®. The knees were not tested in valgus for the same reason.

## CONCLUSION

The present work confirms that the three‐bundle reconstruction of the medial side of the knee, including the sMCL, an AM strand more oblique, and the POL, improves stability in ATT and ER, but with some residual laxity. There is a trend toward better stability with an AM strand more oblique, which must be confirmed by further biomechanical studies. Flat, ribbon‐like MCL grafts represent a biomechanically and anatomically refined approach: they replicate the native ligament's broad shape, distribute forces more naturally, and increase tissue contact for healing. These biomechanical results require to be validated by clinical studies following these principles for reconstruction of severe medial injuries.

## AUTHOR CONTRIBUTIONS

Antoine Hamon and Harold Common contributed equally to the conception, execution, and analysis of the study. Théo Cojean provided biomechanical expertise and statistical analysis. Henri Robert instigated the project and coordinated the research protocol. All authors wrote, read and approved the final manuscript.

## CONFLICT OF INTEREST STATEMENT

The authors declare no conflict of interest.

## ETHICS STATEMENT

All procedures were conducted using donated cadaveric specimens with written donor consent for research and education. The study was performed at the Anatomy Laboratory of the Medical School under institutional oversight. No IRB approval was required as no living human subjects were involved.

## Data Availability

The data sets generated and/or analysed during the current study are available from the corresponding author upon reasonable request.
